# A chromosome-scale fishing cat reference genome for the evaluation of potential germline risk variants

**DOI:** 10.1038/s41598-024-56003-7

**Published:** 2024-04-05

**Authors:** Rachel A. Carroll, Edward S. Rice, William J. Murphy, Leslie A. Lyons, Francoise Thibaud-Nissen, Lyndon M. Coghill, William F. Swanson, Karen A. Terio, Tyler Boyd, Wesley C. Warren

**Affiliations:** 1 Bond Life Sciences Center, University of Missouri, 1201 Rollins St., Columbia, MO 65211 USA; 2 Department of Veterinary Integrative Biosciences, Texas A and M University, College Station, TX 77843-4458 USA; 3 Department of Veterinary Medicine and Surgery, College of Veterinary Medicine, University of Missouri, Columbia, MO 65211 USA; 4 National Center for Biotechnology Information, National Library of Medicine, National Institutes of Health, Bethesda, MD USA; 5 Bioinformatics and Analytics Core, University of Missouri, 1201 Rollins St., Columbia, MO 65211 USA; 6 Center for Conservation and Research of Endangered Wildlife, Cincinnati Zoo and Botanical Garden, 3400 Vine St., Cincinnati, OH 45220 USA; 7 Zoological Pathology Program, University of Illinois, 3300 Golf Rd, Brookfield, IL 60513 USA; 8 Oklahoma City Zoo and Botanical Garden, 2000 Remington Pl., Oklahoma, OK 73111 USA; 9 Department of Surgery, Bond Life Sciences Center, Institute of Data Science and Informatics, University of Missouri, 1201 Rollins St., Columbia, MO 65211 USA

**Keywords:** Genome assembly algorithms, Cancer genomics, Zoology, DNA sequencing, Conservation genomics

## Abstract

The fishing cat, *Prionailurus viverrinus*, faces a population decline, increasing the importance of maintaining healthy zoo populations. Unfortunately, zoo-managed individuals currently face a high prevalence of transitional cell carcinoma (TCC), a form of bladder cancer. To investigate the genetics of inherited diseases among captive fishing cats, we present a chromosome-scale assembly, generate the pedigree of the zoo-managed population, reaffirm the close genetic relationship with the Asian leopard cat (*Prionailurus bengalensis*), and identify 7.4 million single nucleotide variants (SNVs) and 23,432 structural variants (SVs) from whole genome sequencing (WGS) data of healthy and TCC cats. Only *BRCA2* was found to have a high recurrent number of missense mutations in fishing cats diagnosed with TCC when compared to inherited human cancer risk variants. These new fishing cat genomic resources will aid conservation efforts to improve their genetic fitness and enhance the comparative study of feline genomes.

## Introduction

The fishing cat (*Prionailurus viverrinus*) phylogenetically is part of the Leopard Cat lineage, which consists of the Asian leopard, flat-headed, rusty-spotted, and Pallas cats^1^, inhabits the wetlands of Southeast Asia, and, unlike most other felines, relies heavily on waterways for food^2^. Although primarily piscivorous, fishing cats are opportunistic nocturnal predators who also feed on small mammals, amphibians, reptiles, crustaceans, and birds^3,4^. With its stout muscular body, elongated head, webbed paws, and a shortened tail, the fishing cat is well adapted for its aquatic lifestyle. Although habitat loss is the greatest threat to fishing cats^5^, studying the genetics of managed populations can provide insight into their wild ancestry, overall genetic fitness, and facilitate future disease investigations^6,7^.

For many species, maintaining healthy zoo populations is a multifaceted challenge that should include an understanding of the genetics of disease risk. Historically, lethal diseases imperil the sustainability of managed populations and potential releases and thus it is critical to determine the underlying cause^6,7^. Species Survival Plans (SSP) for numerous at-risk species, including the fishing cat, help coordinate breeding efforts to maximize genetic diversity from limited gene pools^6^. In North American zoos, the first appearance of fishing cats can be traced back to founder individuals in the early 1900s, but the majority were imported in the 1960s and 2000s from Sri Lanka, Thailand, and Cambodia. There are currently 25 captive-born individuals carefully managed under an SSP at various accredited institutions where the appearance of diseases with possible genetic causes is especially concerning.

Between the years 1995 and 2004, transitional cell carcinoma (TCC), a form of bladder cancer, accounted for 13% of all zoo-managed fishing cat deaths^8^. TCC has been described in multiple species, including cattle, dogs, cats, some marine mammals, and humans^3,8^ yet its exact cause remains unknown. Although TCC is the most common lower-urinary tract cancer in dogs, it is rare in domestic cats^9^, with the most common clinical sign being persistent hematuria (blood in the urine). TCC tumors in fishing cats occur most commonly in the trigone region of the bladder^8^, consistent with tumor location in the domestic dog and cat^9^. For zoo-managed fishing cats, which are known based on available pedigrees to be highly related, it is possible that the high rates of this cancer are genetic in origin^8^. Risk alleles for TCC have been identified in humans, suggesting a genetic component to the disease^10^. Given this finding of inherited risk in humans, a genome-wide study of all variant types putatively conferring risk is warranted in the fishing cats. These candidate genes can also be evaluated in other species susceptible to TCC, but a highly contiguous reference assembly was previously unavailable for fishing cat.

Highly contiguous genome assemblies, some with gap-free chromosomes, are now becoming readily available for both human and non-human species^11–13^. The currently available fishing cat assembly, PriViv1.0, is highly fragmented with 142,198 total contigs, none of which are assigned to chromosomes, presenting challenges for whole-genome alignment, protein-coding gene completeness, and sequence variant-calling^14^.

In this study, we primarily focus on evaluating previously identified bladder cancer candidate genes^10^. We also expand our search to a recently discovered set of 152 genes associated with inherited cancer susceptibility, both derived from human cancer risk studies^15^. These human germline risk variant profiles collectively allow for better genetic risk assessment of captive fishing cats. We present a new high-quality fishing cat reference genome, the most complete pedigree of captive fishing cats currently available, and whole genome sequencing (WGS) data from 11 cats with and without TCC, as well as their called single nucleotide variants (SNVs) and structural variants (SVs). With this new catalog of discovered sequence variants, we enable future studies that attempt to understand this type of bladder cancer and its occurrence in other species. In addition, we examine the assembled accuracy of this fishing cat reference by comparing genome synteny to a closely related cat species, the Asian leopard cat.

## Results

### Pedigree construction

**Figure 1:**
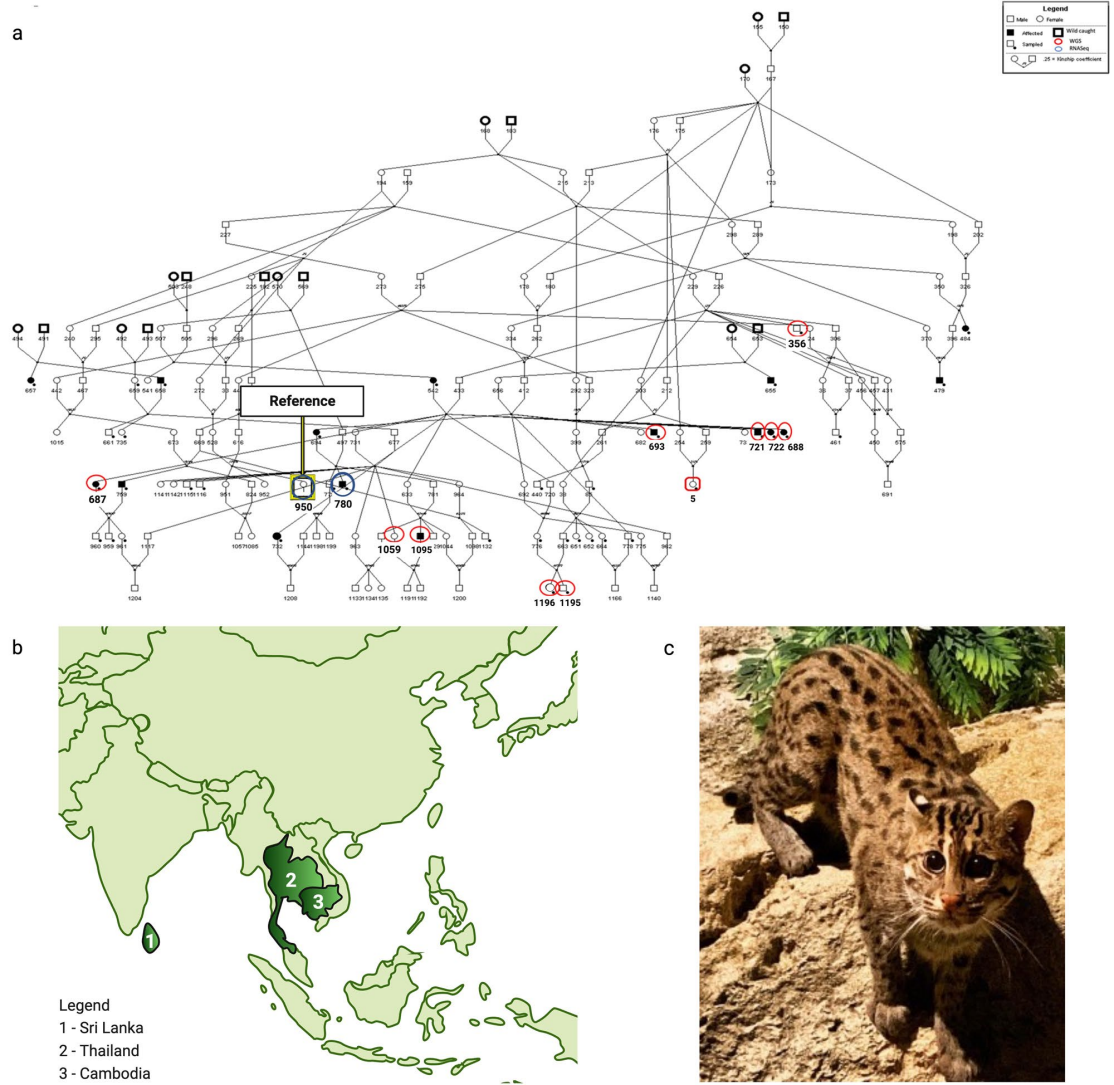
The pedigree and geographic origins of founder individuals. (**a**) North American fishing cat population pedigree. In total there are 161 cats in the historic population, with 25 of those being cats currently in zoo-managed care and 15 total cats being confirmed as TCC cases. (**b**) Geographical origins of the current North American zoo fishing cat population individuals according to studbook information. Of the 16 founder cats, 6 were from Sri Lanka, 5 from Thailand, and 2 from Cambodia. The remaining 3 were documented as being from Asia, however, no specific country of origin is indicated. (**c**) Pictured here is the fishing cat selected for the reference genome assembly. Anna (AZA Studbook #950). Initially born in the La Fleche Zoo on 9/14/2010. After being transferred to two other European facilities in Bucharest, Romania, she eventually joined the AZA population in 2010 at the Chicago Zoological Society. She was then transferred to the Oklahoma City Zoo in 2020. Photograph provided by Animal Care Specialist Natalie Farley, Chicago Zoological Society. Figure adapted from “BioRender Templates—Create New”, by BioRender.com (2023). Retrieved from https://app.biorender.com/biorender-templates.

The historic population in North American zoos is comprised of a total of 161 cats (Fig. 1a). By reconstructing this pedigree, we could identify individuals that would be most informative for investigating germline alleles potentially associated with TCC occurrence. Using the 2019 fishing cat studbook, each living cat was traced back over 12 generations to the founding individuals and their respective origins, primarily from the countries of Sri Lanka, Thailand, and Cambodia (Fig. 1b). Some founders were labeled as Asia origins, but we could not establish a country of origin. Although there are only 25 cats currently managed within in the Association of Zoos and Aquariums (AZA), the historical SSP population numbered as many as 60 cats. From this cohort, we were able to identify a reference individual (Fig. 1c), as well as all cats selected for WGS and bulk RNA sequencing (RNAseq) to investigate TCC occurrence.

The completed pedigree reveals multiple offspring resulting from consanguineous mating. Any consanguineous mating occurring between second cousins or closer was identified for the purposes of this study^16^. Some examples included in the Studbook (SB) are animal 226 mated with 229 (full sib to 226’s dam) to produce offspring 431, 433, 37, 456, 306, 356, and a full-sib mating pair of 175 and 176 that produced 213, 212, 298 and 254 at the top right of the pedigree (Fig. 1a). Many of these closely related individuals went on to produce at least one fishing cat in the pedigree. On the right side of the pedigree the founder cats 168 (Thailand), 183 (Thailand), and 170 (Sri Lanka) were all born between the 1970s-80 s and were initially bred and managed outside of North American zoos. Additional founders on the left side of the pedigree, including 491–494, 182 (Sri Lanka), 503, and 569-570 (Thailand), were all born in the 1980s and 1990s. In the early 2000s, two additional founders in the middle of the pedigree, 653-654 (Cambodia), were introduced. The number of consanguineous mating events fortunately occurred in earlier generations with later changes implemented by the AZA and SSP groups that reduced inbreeding.

### De novo* assembly*

An SSP 11 year-old female, Anna (AZA studbook #950, Fig. 1c), from the Oklahoma City Zoo, was chosen for long-read sequencing and genome assembly. At the time of death, Anna was determined to be TCC negative following a necropsy. We generated 30 × sequence coverage of PacBio HiFi reads and assembled these into 245 primary contigs using HiFiasm^17^. Contig scaffolding was accomplished using ~ 35 × sequence coverage of a Hi-C library^18,19^ in 19 chromosomes and 172 unplaced individual contigs or scaffolds (Supp. Figure 1a, b). Manual visualization of MashMap^20^ alignments against the domestic cat (Supp. Figure 2a, b)^21^ and Asian leopard cat^12^ assemblies verified chromosome orientation (Supp. Figure 3). The total assembled size was 2.46 Gb with N50 contig and scaffold lengths of 68.7 Mb and 144.9 Mb, respectively, with 96.3% of sequence assigned to chromosomes. These assembly metrics are similar to the single haplotype assemblies of domestic cat and Asian leopard cat genomes^12^ (Table 1). In contrast, compared to the prior short-read-based fishing cat assembly (PriViv1.0)^14^ our reference increased in size by 16 Mb and N50 contig length by 2000 fold.

**Table 1:** Summary statistics for select feline reference assemblies.

	Domestic cat (F.catus_Fca126_mat1.0)	Asian leopard cat (Fcat_Pben_1.1_paternal_pri)	Fishing cat (UM_Priviv_1.0)
Total ungapped bp length	2,425,739,938	2,435,706,361	2,461,531,252
Number of scaffolds	71	84	192
Scaffold N50 bp length	148,491,486	148,587,958	144,906,162
Number of contigs	110	140	255
Contig N50 bp length	90,731,473	82,622,880	68,766,634

Summarized are the assembly statistics for the following species: domestic cat, Asian leopard cat, and fishing cat.

### Genome annotation

Benchmarking Universal Single Copy Orthologs (BUSCO) analysis was performed to assess genome completeness^22^. We find 93.5% of orthologs were complete, with 4.7% missing compared to 5.0% in Asian leopard cat^12^ (Supp. Table 1). BUSCO^22^ results were consistent with other highly contiguous feline genome assemblies (Supp. Table 1). To validate in silico gene predictions, we generated RNAseq data for two fishing cat individuals, Kiet (TCC positive at time of death; animal identification SB #780) and reference cat Anna (TCC negative at time of death; animal identification SB #950), from bladder and kidney tissue, respectively. Using the standardized NCBI RefSeq genome annotation processes^23^ (see Supp. Table 2 for the complete NCBI annotation report) resulted in 20,055 and 6904 predicted protein-coding and non-coding genes, respectively, similar to estimated total gene counts of other feline species (Supp. Table 3).

### Genome synteny analysis

**Figure 2:**
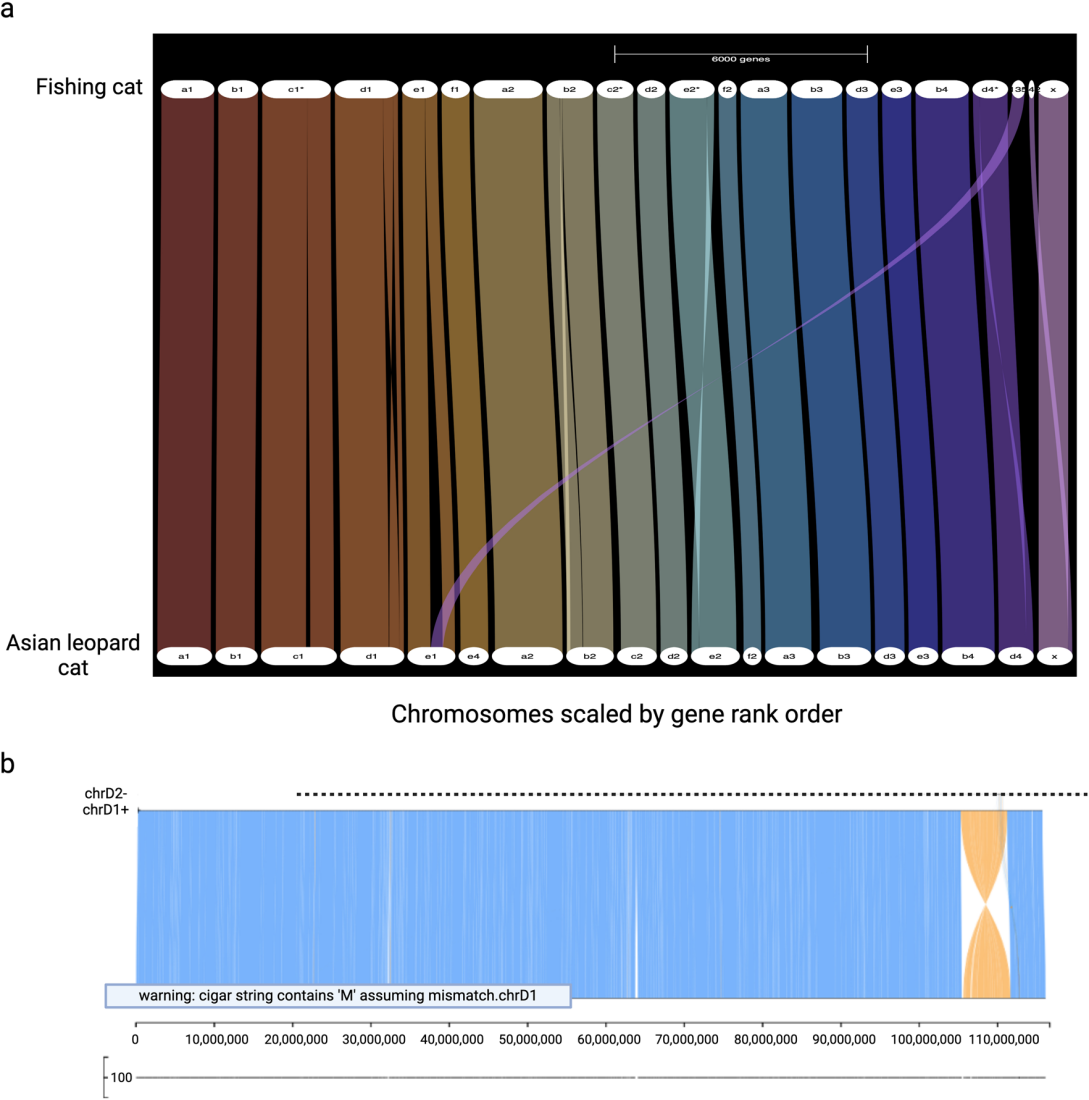
Chromosome alignments between the Asian leopard cat and fishing cat. (**a**) Synteny plot comparing the Asian leopard cat chromosomes (ALC) to the fishing cat chromosomes (FC). Most alignments pictured are found to exhibit a high level of synteny with each other. (**b**) Examining alignments between the Asian leopard cat and fishing cat, the chromosome with an interesting alignment is in D1 as indicated by this SafFire^26^ output. D1 alignments illustrate an inversion ~ 6.0 Mb in size towards the end of the alignment.

Cross-species whole genome comparisons between recently diverged species can highlight signatures of evolutionary adaptation and speciation but also misassemblies. The large-scale sequence structural similarities and differences between the fishing cat and Asian leopard cat assemblies Fcat_Pben_1.1_paternal_pri^12^, which diverged from fishing cat ~ 3 million years ago^24^ were measured to ensure assembly accuracy using two independent approaches Genespace^25^ and SafFire^26^. Overall, manual reviews of whole genome alignments confirmed an expected high amount of one-to-one chromosomal synteny (Fig. 2a), but one obvious distinction was a large putative inversion (6 Mb) toward the end of chrD1 (Fig. 2b). The chromatin proximity map shows the highest probability supporting its current fishing cat inverted orientation and their breakpoint boundaries reside in an un-gapped region of the assembly that each suggest this inversion to be a natural difference to the Asian leopard cat. However, further confirmation in additional fishing cat genomes will be needed to be certain.

### Cohort sequencing and variant annotation

**Figure 3:**
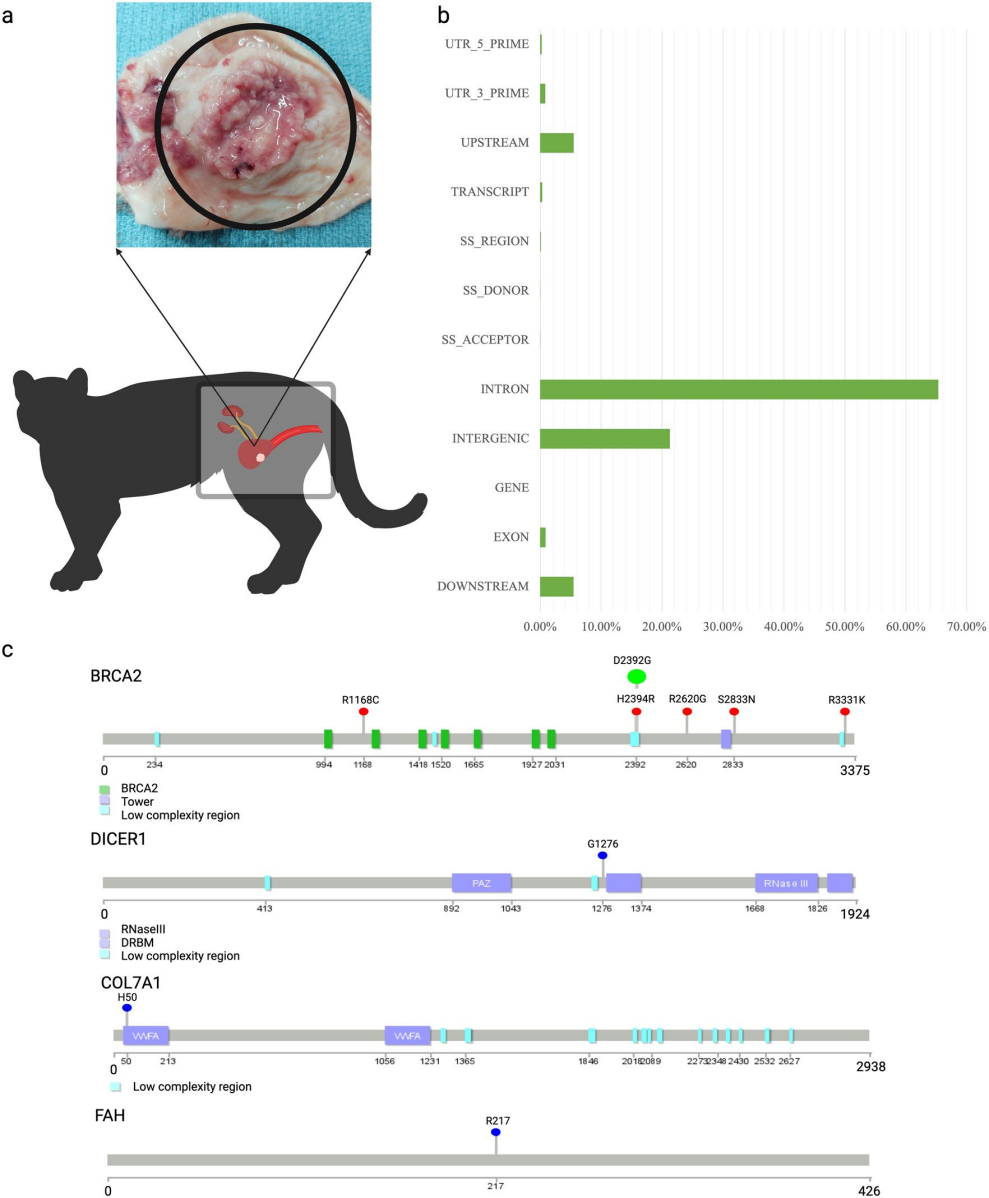
Missense variants identified in the fishing cat cohort. (**a**) A fishing cat urinary tract. Indicated by the black circle is a tumor excised from the bladder wall of a transitional cell carcinoma affected fishing cat. Tumor photograph provided by Dr. Leslie Lyons, University of Missouri. (**b**) Depicted in this bar graph are the variant types identified in the fishing cat cohort. The most prevalent were found to be intronic and intergenic variants, representing 65.31% and 21.28% respectively. Additionally, downstream variants and upstream variants were also present at 5.48% and 5.51%. Lastly, additional variants such as exon, gene, splice site acceptor, splice site donor, transcript, UTR 3 prim, and UTR 5 prime collectively comprised of 2.43% of all variants identified. (**c**) This lollipop plot illustrates six of the missense locations present in all cancer cats in *BRCA2* (LOC125162678) and the single missense positions in *DICER1* (LOC125167827), *COL7A1* (LOC125159913), and *FAH* (LOC125158967). All small red lollipops on all genes indicate positions more strongly correlated with cancer cats. For *BRCA2* all cancer cats expressed missense mutations at all indicated locations, with some normal-presenting cats also experiencing missense variants. D2392G on *BRCA2* is indicated by a larger green lollipop as the heterozygous genotype is unique to only cancer cats. At the five other *BRCA2* positions 40% of all control cats are also affected. Position G1276 on *DICER1* shows 83% of cancer and 20% of control affected. Position H50 on *COL7A1* shows 100% cancer 60% control cats affected. Position R217 on *FAH* shows 83% cancer and 20% of control cats affected. All sites on all genes experienced an amino acid change because of the base pair changes. Figure adapted from “BioRender Templates-Create New”, by BioRender.com (2023). Retrieved from https://app.biorender.com/biorender-templates.

Over the past 15 years, 15 fishing cats were diagnosed with TCC that were each verified by veterinarian pathology reports and histologic confirmation of the bladder wall tumor (Fig. 3a). In total, based on sample availability we generated WGS data on 11 cats (six TCC and five unaffected; Supp. Figure 4 and Supp. Table 4) each at ~ 25 × sequence coverage on average to detect SNVs and indels. The Genome Analysis Tool Kit (GATK)^27^ was used to detect an initial set of 7,541,694 SNVs and 2,600,943 indels (1-291 bp in size). This initial set was further filtered to reduce the presence of false positive SNVs and indels by choosing an optimal plot inflection in the quality depth (QD) parameter at < 5 as a cutoff for both SNVs and indels. A final hard-filtered total of 7,431,632 biallelic SNVs and 2,453,263 indels was obtained. In addition, a total of 10,704 multiallelic SNVs were observed but not considered in further analyses.

To generate a comprehensive catalog of SNVs and indels that are characterized by their predicted impact on protein function, SnpEff^28^ was run. The total variants composition used to predict impact was 68.00% SNVs, 15.09% insertions, and 16.91% deletions (Fig. 3b). As seen in other studies^21,29^ of variant classification, nonsense, missense and silent were identified. Nonsense, or loss of function (LOF), variant counts were rare at 0.299% (n = 508). Missense (amino acid altering) and silent (nucleotide variation with no amino change) variants accounted for 40.044% (n = 68,113) and 59.657% (n = 101,473) of the total, respectively. A summary of all variant types that fall into these three broad categories are specified in Supp. Table 5, with variant rate across chromosomes detailed in Supp. Table 6.

### Cancer candidate gene screening

A list of 152 known cancer risk genes^15^, which includes ten bladder cancer risk genes previously identified in human cancer patients^10^ were examined for orthologs in the fishing cat cohort. Due to the lack of research into characterizing the genetics of TCC in fishing cats, a candidate gene strategy was implemented by searching for inherited LOF or missense variants in the fishing cat gene orthologs of first the ten human bladder cancer risk genes that included *BRCA1, BRCA2, CHEK2, ATM, MSH2*, *MUTYH*, *MITF*, *MLH1, FH,* and *FANCC*^10^ then a larger set 152 cancer risk gene set^15^. Of all ten bladder risk candidate genes, only *BRCA2* demonstrated higher missense variant presentation in TCC cats compared to unaffected cats (Fig. 3c) and at all positions (R1168C, H2394R, R2620G, S2833N, R3331K, D2392G), TCC cats expressed a heterozygous genotype. However, D2392G was the only genotype uniquely shared among all TCC cats and none of the unaffected individuals (Supp. Figure 5).

When evaluating fishing cat gene orthologs among the 152 inherited cancer driver genes listed^15^ a total of 107 genes were identified as orthologous and further examined for higher TCC occurrence. No TCC cats displayed LOF variants in these genes, but a higher prevalence of missense variants was seen in four genes*: BRCA2, COL7A1, DICER1*, and *FAH* (Table 2; Fig. 3c).

**Table 2:** Pathogenic risk genes and associated missense variants per gene.

Symbol	Fishing cat NCBI gene ID	Chromosome	Chromosome variant base position	REF	ALT	Variant type
*BRCA2*	LOC125162678	A1	NC_062561.1:11545118	C	T	missense
			NC_062561.1:11559028	A	G	missense
			NC_062561.1:11559034	A	G	missense
			NC_062561.1:11565330	A	G	missense
			NC_062561.1:11571359	G	A	missense
			NC_062561.1:11587856	G	A	missense
*COL7A1*	LOC125159913	A2	NC_062562.1:17133400	G	A	missense
*DICER1*	LOC125167827	B3	NC_062566.1:133534186	C	G	missense
*FAH*	LOC125158967	Scaffold	NW_025927608.1:3060456	C	T	missense

Indicated are the human gene IDs, fishing cat LOC IDs, chromosome, variant position, reference allele, alternate allele, and variant type. In total, *BRCA2* had the most (6) missense variant positions identified.

### SV discovery

**Figure 4:**
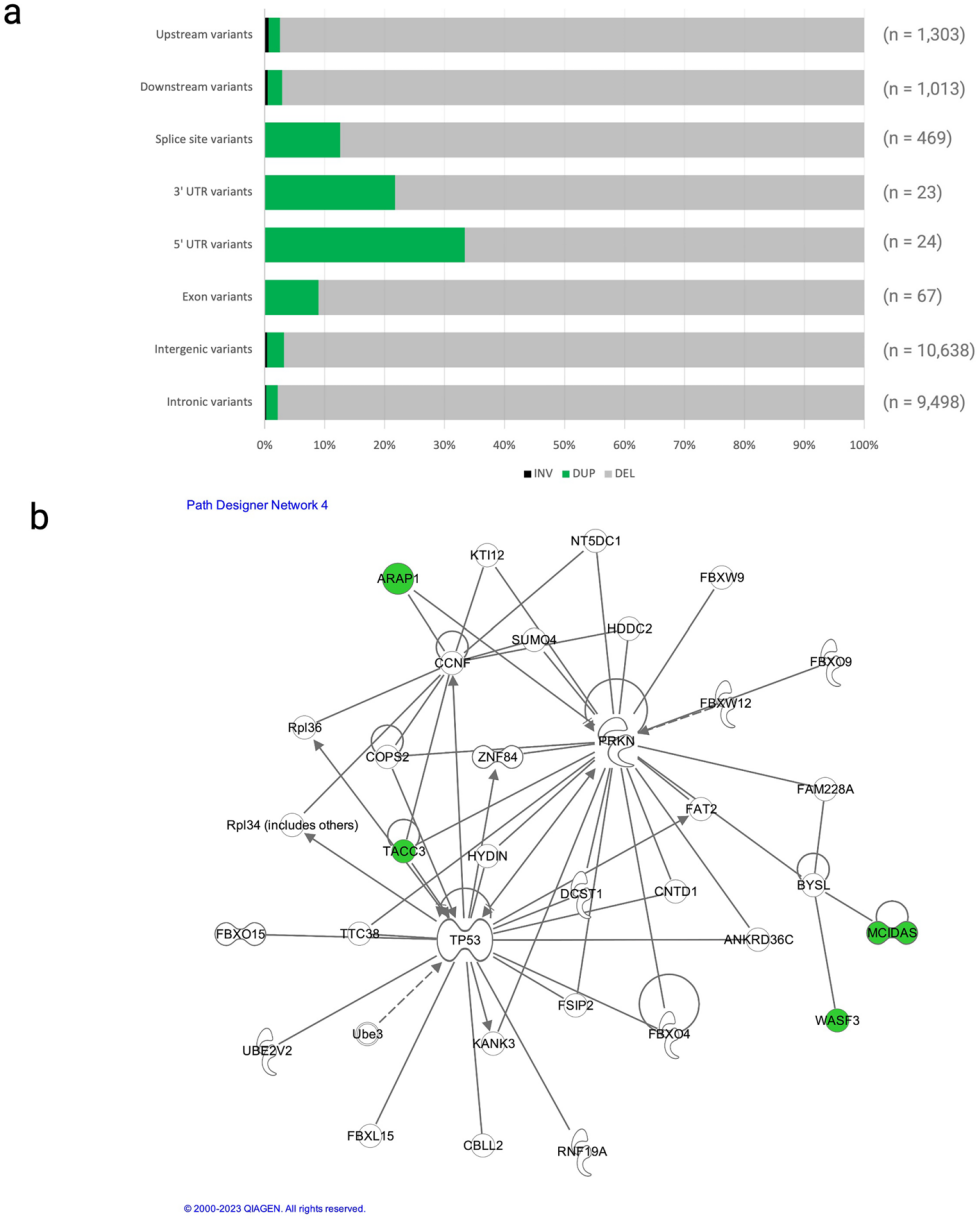
Structural variant analysis of coding regions. (**a**) This stacked bar plot depicts the classification of SV types (deletions, insertions, and duplications in the following genomic regions: upstream, downstream, splice site, 3’ UTR, 5’ UTR, exon, intergenic, and intronic regions. Of the total 23,432 SVs, 22,419 were classified as deletions, 910 duplications, and 103 inversions. Of the variant classifications, intergenic and intronic were the most prominent with 10,638 and 9498 being identified respectively. (**b**) This tumorigenesis pathway includes four genes (highlighted in green) containing coding region SVs present in the cohort. At least one cancer cat had to contain the SV to be examined in this analysis. Of the four genes, two that were only present in cancer cats were *MCIDAS* and *ARAP1.*

To discover the scope of SV diversity segregating in fishing cats and investigate their possible risk association among inherited cancer risk genes, we genotyped SVs in all sequenced cats using Lumpy^30,31^ and SnpEff^28^. A total of 23,432 SVs were discovered, including 22,419 deletions, 910 duplications, and 103 inversions were identified. Deletions were examined for their possible effect on protein-coding genes as in Warren et al.^31^ (Table 3; Fig. 4a). Only deletions were further examined due to their overall predominance in this cohort. No germline cancer risk genes evaluated in this study contained deletions that occurred predominantly in TCC cats^15^. However, a search beyond this candidate gene set found six deletions as having unique shared genotypes in only TCC cats with five in intronic regions of the following genes: *DOCK4, TRIT1, CSMD2, CDV3,* and *B4GALNT2.* For *DOCK4,* all TCC cats shared a homozygous intronic deletion compared to all unaffected cats, which were heterozygous for this deletion. For the remaining four genes, all TCC cats shared a heterozygous deletion, while unaffected individuals did not have this deletion in either haplotype. One deletion (238 bp) was found in the regulatory region upstream of the *SMIM30* gene as defined by±2000 bp proximal or distal to the intronic or coding region of a gene (Supp. Table 7)*.*

**Table 3:** Total structural variants identified per individual fishing cat.

Structural variant type	Fritz (SB356)	Splash (SB05)	SB1195	Juniper (SB1196)	Jonas (SB1059)	Padma (SB688)	Pavarti (SB687)	Gorton (SB721)	Maliha (SB722)	Sushi (SB693)	Wasabi (SB1095)
Deletion	9837	12,971	9745	10,437	10,717	9036	10,336	10,333	8684	10,907	8704
Inversion	27	18	23	21	23	27	26	26	18	21	19
Duplication	151	151	123	139	113	105	132	157	113	162	107
Total	10,015	13,140	9891	10,597	10,853	9168	10,494	10,516	8815	11,090	8830

Above outlines the SV type and count per fishing cat individual. All SVs identified are between 50 and 10,000 bp in size. Cats diagnosed with TCC are Padma, Pavarti, Gorton, Maliha, Sushi, and Wasabi.

The potential impact of protein-disruptive SVs on pathways associated with tumor formation was also examined using the QIAGEN Ingenuity Pathway Analysis (IPA) software^32^. Of a total 104 SVs ranging from 50 to 10,000 bp in size, 44 had associated gene ontology that allowed us to test disease function enrichment. In total, two tumor-associated signaling pathways, one with *TP53* as the central hub, were explored (Fig. 4b; Supp. Figure 6). Eight genes were identified with SVs occurring in at least one cancer cat, with two genes found to impact only cancer individuals: *ARAP1* and *MCIDAS* (Fig. 4b). *ARAP1* plays an important role in cellular apoptosis^33^, and *MCIDAS* is involved in both multiciliate cell differentiation as well as cell cycle exit during mitosis^33^. Cancer cats Pavarti, Sushi, and Wasabi shared a coding sequence deletion 530 bp in size in *ARAP1,* and Wasabi, Pavarti, and Gorton shared a coding sequence deletion 1967 bp in size in *MCIDAS.*

## Discussion

A pedigreed fishing cat female was used to generate a chromosome-scale reference with a 2000-fold improvement in continuity compared to the previous scaffold-level assembly, Priviv1.0^14^. The overall assembly quality metrics were comparable to the two phased assemblies derived from an F1 Bengal cat hybrid: domestic cat and Asian leopard cat^12^. These sequence completeness and accuracy measures demonstrate that this new fishing cat reference is the optimal computational resource for investigating genetic risk factors for diseases in the fishing cat, as well as its population diversity and new hypotheses raised regarding Felidae interspecies genome evolution^34^.

Various measures of gross co-linearity indicate that Felidae genomes are highly conserved for most chromosomes^35,36^. Within the felid phylogeny, the fishing cat is closely related to the Asian leopard cat^1^, which prompted us to search for any major structural changes that have occurred within the past 3 Myr since their divergence. We confirmed substantial conserved genome-wide synteny in these two *Prionailurus* species using a variety of alignment techniques. This result is consistent with the high level of genomic synteny at the moderate and deepest divergence of the cat family demonstrated in comparative studies of the domestic cat^12,37^. Nonetheless, many interesting small-scale and even rare large deviations in sequence order and orientation have arisen within the *Prionailurus* genus, such as a putative 6 Mb inversion on chromosome D1 that we discovered. It is plausible to hypothesize some of these structural differences, although beyond the focus of this study, are contributing to unique, lineage-specific felid species phenotypes^12^.

Accurate pedigrees including disease state are important tools for avoiding the propagation of risk alleles for diseases, such as some cancers, that appear after reproductive age in small and inbred zoo-managed populations. Our construction of the largest fishing cat pedigree to date, 161 individuals, is a first step in investigating the increased incidence of TCC observed in captive fishing cats, as there are only 25 captive-bred individuals remaining in North American zoos today.

Although no genetic cause or mode of inheritance has been found, the clinical symptoms of bladder cancer in captive fishing cats were first observed as far back as 1991 and later proposed to be TCC^3^. However, its occurrence throughout the pedigree led us to further genetic evaluation. Our study provides the first estimation of segregating SNVs, indels and SVs in fishing cats and their potential use for investigating disease origins. Of the missense variants examined in fishing cat gene orthologs for human bladder cancer risk genes, DNA damage repair pathway genes *BRCA1/2, CHEK2,* and *ATM* all showed higher missense variant prevalence in the cohort; however, only *BRCA2* showed a skewed distribution in TCC over healthy cats. In humans, *BRCA2* variants long known for their roles in familial breast, ovarian, pancreatic, and prostate cancers^38–40^ have more recently also been connected to predisposition for some types of human bladder cancer^10,39,41^. Moreover, increased numbers of germline variants associated with DNA damage repair pathways in human urothelial carcinoma (UC) patients (e.g. *BRCA1/2, CHEK2,* and *ATM*), particularly in *BRCA2*^10,41^ have led one group to call for *BRCA2* germline UC screenings^41^.

When investigating the larger set of 107 inherited genes conferring risk across cancer types^15^, three additional candidate genes containing variants with higher prevalence in TCC cats were identified: *DICER1, FAH,* and *COL7A1.* Interestingly, the same heterozygous genotype for sibling cancer cats (Maliha, Padma, Pavarti, Gorton) was shared in both *DICER1* and *FAH. FAH* and *COL7A1* have not previously been associated with bladder cancer in human; however, mutations within these genes are connected to other cancers. In humans, *FAH* is typically associated with the liver disorder Hepatorenal Tyrosinemia Type 1, which can result in liver cancer^42^. *COL7A1* is a type VII collagen associated with collagen production in epithelial cells, with mutations previously linked to the development of skin cancer^43^. Upregulation of *COL7A1* has also recently be identified in patients with gastric cancer^44^. Unlike *FAH* and *COL7A1, DICER1* has been directly investigated for its role in bladder cancer patients^42^. Since *DICER1* is a tumor suppressor gene and found to be previously downregulated in human TCC^42^ we propose that its role in fishing cat TCC occurrence merits further study.

Because it is more difficult to genotype SVs as opposed to SNVs and short indels, few germline SVs with disease associations have yet been found in any felid species^45,46^. The SVs discovered herein are the first for fishing cats and dissimilar to totals found in other cats^21,29^. We found fewer total SVs in fishing cats compared to other cats such as domestic cats^21^ and tigers^29^, likely because of the close genetic relatedness to the reference cat of the re-sequenced cats in this study, a result of inbreeding in zoo-managed populations. Like the domestic cat, most SVs we identified are commonly shared, suggesting their impacts are mostly tolerated^21^.

Some of the eight deletion-affected genes we found have human orthologs with variants documented in various cancer types^47–51^. Examples include *SMIM30* in hepatocellular carcinoma^47^ and *CDV3,* in breast^49,51^ and colorectal adenosarcoma cancers^48,49^ In a recent pan-cancer study, *CSMD2* was identified in 25 of 33 cancer types, with the highest expression found in gastric, lung, colorectal, and prostate cancer^50^.

The importance of SVs to cancer occurrence overall is deservingly receiving more attention as their broader affect is underappreciated due to their specificity by cancer type being greater than SNVs^46^. However, at present the small number of fishing cat SVs that were predicted to alter protein function and importantly impact previously implicated germline risk genes in bladder or other cancer types prevents us from suggesting screens for any fishing cat genes as a result of SV disruption.

The sequence characterization of all potentially deleterious variants, whether single base or structural, segregating in fishing cats, highlights the importance of, in conjunction with building accurate pedigrees, assessing the risk that small captive populations face. This is particularly crucial as they are often started with very few founder individuals. The comparative depth of knowledge available for a sequence variants impact in human is particularly helpful to guide species survival program, specifically mating decisions to genetically alleviate future disease occurrence. We suggest these genome-wide findings in captive fishing cats will better illuminate their genetic fitness, with a goal to diminish the occurrence of any diseases in this small fragile population, thus promoting their future conservation.

## Methods

### Pedigree construction

A pedigree was drawn using the program Pedigree-Draw (version 6.0, March 2005, Jurek Software) to select the reference individual that accurately represents the Association of Zoos and Aquariums (AZA) population. Using the current fishing cat studbook from 2019, each living cat was traced back over 12 generations to the founding individuals and their respective origins.

### Reference individual

Anna, an 11 year-old female fishing cat from the Oklahoma City Zoo, was selected for genome assembly and her position in the pedigree has been documented. High molecular weight (HMW) DNA was obtained from frozen kidney tissue extracted during necropsy and stored at − 80 °C. The HMW DNA was isolated using the 10 × Genomics Demonstrated Protocol: DNA Extraction from Single Insects (10 × Genomics). The final HMW DNA quantity was determined using the high-sensitivity Invitrogen Qubit Fluorometer protocol. Final HMW quality was determined using a 0.7% agarose gel and imaged on the Uvitec Cambridge Uvidoc HD6 UV Fluorescence and Colorimetry instrument.

### Genome long-read sequencing

Isolated HMW DNA was used for library construction and long-read sequencing. Small fragment removal from HMW DNA on a Blue Pippin Instrument (10–50 kb size range) was done before shearing using a megaruptor shear speed 31. A 20 kb fragment size cutoff was used to construct SMRTbell libraries using the CCS Express Library Kit V2. The final library concentration was 38.6 ng/ul from which three SMRT cells were generated on a Sequel II instrument using HiFi mode (PacBio) to an estimated 30 × genome coverage of highly accurate circular consensus sequences (CCS).

### Assembly construction and curation

De novo assembly used CCS processed reads > 18 kb and was performed with Hifiasm (version 0.13–2208)^17^. BUSCO (version 4.1.2_cv1)^22^ analysis with the arguments “-m genome –l mammalia_odb10” was used to estimate genome completeness^12^. To reduce redundancy due to assembled heterozygous sequences the purge_haplotigs pipeline^52^ was run^53^ but no distinct haplotig curve was observed in the histogram therefore no contigs were removed^52^. BUSCO^22^ confirmed a low gene duplication rate and level of redundant sequences. To scaffold the assembly, R1 and R2 Hi-C reads generated by the DNA Zoo^18,19^ were aligned with bwa v0.7.17^54^. Following sequence post-alignments processing a series of custom python scripts and established tools were run to filter chimeras and combine the R1 and R2 read alignments including: samtools v1.9^55^ commands “fixmate”, “sort”, and “markdup” to fix the mate pairs, sort the alignments by position, and mark duplicates, respectively. We converted the alignments to bed format using bedtools v2.27.1^56^ “bamtobed”. Finally, we ran Salsa (version 2.2) and Juicebox (version 1.11.08)^57^ to evaluate the resulting Hi-C heat maps for order and orientation convergence. The pipeline and custom scripts written for this purpose can be found in the Code Availability section.

Misplaced scaffolds were identified using multiple programs and orthogonal evidence to estimate the most accurate chromosome order and orientation. Chromosome nomenclature was assigned in accordance with the original domestic cat genetic linkage map groupings^58^. MashMap (version 2.0)^20^ was used to compare the fishing cat pseudochromosomes to the domestic cat reference Felis_catus_9.0^21^ to aid in the detection of misassembles. Using both Juicebox^57^ and MashMap^20^ output, chromosomal accuracy benchmarking analysis identified 37 chromosomal scaffolds requiring correction. For example, fishing cat scaffold_4 covered the entirety of domestic cat chrB2 (Supp. Figure 2a). Yet contradictory alignment evidence led us to break this scaffold into three pieces and rejoin each in the correct order and orientation. Reevaluation with the MashMap^20^ and Hi-C contact maps confirmed their accuracy. If local order within a contig was not conserved, we avoided homogenizing the fishing cat assembly to mirror the domestic cat thus preserving the original fishing cat genome structure whenever orthogonal evidence supported it (Supp. Figure 2b). Agptools was used to finalize chromosome assignments to be consistent with the linkage groups of the domestic cat genome^58^.

### RNA sequencing and gene annotation

Total RNA from the bladder and spleen of two fishing cats was isolated via the RNeasy Plus Universal Mini Kit (Qiagen). The bladder sample was from a 12 year-old male Kiet from the San Francisco Zoo, while the spleen sample originated from an 11 year-old female fishing cat Anna from the Oklahoma City Zoo. The cDNA libraries of each sample were generated and sequenced on an Illumina Novaseq 6000 to a targeted coverage of 50 million reads/library. To verify RNAseq quality the alignment program STAR (version = STAR_2.5.2b)^59^ was used to map both sequences to the reference genome. Reference indices were generated using the fishing cat reference and associated GTF file. RNAseq data for each cat was aligned using standard STAR parameters^59^. Cats Anna and Kiet were found to have sufficient uniquely mapped reads percentages of 72.61% and 78.66% respectively. Both RNA datasets were submitted to the NCBI sequence read archive and used to verify gene predictions in the RefSeq annotation pipeline^23^.

### Genome synteny

Two independent approaches were used in this analysis. An R package tool Genespace (version 1.1.9)^25^ was used to produce a whole genome synteny plot, while SafFire (version 0.2)^26^ allowed for a higher resolution assessment of synteny across individual chromosomes. Cross-species alignments between the fishing cat (UM_Priviv_1.0) and Asian leopard cat (Fcat_Pben_1.1_paternal_pri) genomes^12^ were performed using minimap2 (version 2.24-r1122)^60^ with the asm20 flag and Rustybam (v0.1.30)^61^. This generated alignment file was used as input for SafFire^26^. To optimize program performance, all unplaced scaffolds were removed, and NCBI chromosome nomenclature was changed to the feline nomenclature prior to running Minimap2^60^. The Genespace^25^ riparian plot was generated using the specified parse annotation function to create the gene bed file from the fishing cat and Asian leopard cat assembly and GFF files. Once parsed, the files were then run through Genespace using OrthoFinder (version 2.5.4) and the MCScanX package^62^.

### TCC sample preparation and sequencing

A total of 11 cats with and without presenting TCC were selected for WGS. Biopsies of suspected tumor tissue were performed to confirm the TCC status of affected individuals. DNA was isolated from all samples using either whole blood or tissues with the Qiagen DNeasy Blood and Tissue Kit (Qiagen). The DNA quantity and quality was determined using the Qubit Fluorometer instrument protocol (Invitrogen), and electrophoresis was performed on a 0.7% agarose gel with gel imaging on the Uvitec Cambridge Uvidoc HD6 UV Fluorescence and Colorimetry instrument, respectively. Sequencing libraries were generated using the Illumina DNA prep protocol (Illumina) with the exception that we used double bead selection to obtain larger insert sizes (550 bp). All libraries were sequenced on the Illumina Novaseq 6000 (Illumina) with a targeted 20 × genome coverage per cat.

### Variant call analysis

The Genome Analysis Toolkit (GATK; version 4.1.8.1)^27^ was used for variant identification in a population cohort of 11 fishing cats, both with and without presenting TCC (Suppl. Table 4). GATK was run with default parameters in conjunction with HaplotypeCaller^63^, allowing for the joint genotyping of germline variants in all individuals. The default GATK hard filtering parameters for SNVs and indels were as follows: QD < 2, QUAL < 30, SOR > 3, FS > 60, MQ < 40, MQRankSum < − 12.5, ReadPosRankSum < − 8, and QD < 2, QUAL < 30, FS > 200, ReadPosRankSum < − 20, respectively. To visualize the distribution of SNVs and indels we used gridExtra and ggplot2 modules within RStudio (version 2021.09 + 351). The final QD was adjusted to < 5 for both SNVs and indels with the remaining filters remaining the same. The Nextflow variant call pipeline was then re-run with the new filters and all statistics for the GATK VCF output were obtained using BCFTools stats (version 1.14)^55^.

### Neighbor-joining trees

An unrooted radial phylogram was illustrated to visualize the relationships within the fishing cat cohort. The fishing cat VCF file was converted to phlyip format using Vcf2phylip (version 2.0)^64^ to allow for subsequent phylogenetic analysis. The phylogenetic assessment of the cohort was conducted using the Molecular Evolutionary Genetics Analysis (MEGA) software (version 11)^65,66^. The neighbor-joining method was performed using the nucleotide sequences option, the Kimura 2 Parameter mode to estimate genetic distances between each sample within the phylogenic tree^67^, and the Standard Select Genetic Code option. Additionally, a bootstrap analysis was run with 1000 replicates to evaluate branch correctness, ensuring accuracy of within cohort phylogeny.

### Variant effect and annotation

For genomic variant annotations and functional effect prediction SnpEff (version 5.1d)^28^ and SnpSift (version 5.1d)^68^ were used. The final SnpEff database was generated using reference genome UM_Priviv_1.0 and the NCBI associated protein and gene annotation files^23^.

### Cancer risk gene investigation

A set of 152 cancer risk genes^15^ was analyzed for orthologous protein sequences in fishing cat through OrthoFinder^69^ (version 2.5.4). This analysis used the human, domestic cat, Asian-leopard cat, tiger, and fishing cat genomes. Human-fishing cat orthologous genes were then inferred from the matched protein sequences. In addition, ten human bladder cancer germline risk genes reported in Nassar et al.^10^ were integrated to refine the search for known bladder cancer risk genes^10^. All ten of these genes were encompassed in the larger 152 cancer risk gene dataset^15^. SnpEff^28^ (version 5.1d) was used to generate a VCF classifying variant types per cat in the fishing cat cohort. SnpSift (version 5.1d)^68^ was used to filter variant types based on missense and loss of function (LOF) variants and filtered based on the cancer gene set identified by OrthoFinder. BCFtools^55^ (version 1.16) was used to evaluate the prevalence of missense and LOF variants in the cohort. The Integrative Genomics Viewer (IGV) (version 2.13.2)^70^ was used to identify any variants found within the identified genomic regions using the reference genome and index, GFF, and VCF files with all the population data. All lollipop plots were illustrated using Lollipops (version 1.6.0)^71^.

### Structural variant analysis

We genotyped structural variants in all the short-read cats using lumpy^30^ via the smoove pipeline v0.2.3, as previously described^31^. Briefly, for each cat, we aligned reads to the fishing cat reference using bwa mem v0.7.17^54^ with the argument ‘-R “@RG\\tID:${accession}\\tSM:${accession}\\tPL:ILLUMINA”‘, and subsequently ran the output through the samtools v1.16.1^55^ commands “fixmate -m”, “sort”, and “markdup -r”. We then ran the smoove commands “call”, “merge”, “genotype”, and “paste”, as described in the smoove documentation. The Nextflow pipeline we used to run all these commands is publicly available at^31^. SnpEff (version 5.1d)^28^ and SnpSift (version 5.1d)^68^ were used to annotate the SVs. The stacked bar plot was generated by plotting all SVs associated with the specified genomic regions of interest. Genes with SV-affected coding regions were filtered based on size (50–10,000 bp) and gene annotation status. All identified genes were further evaluated through the QIAGEN Ingenuity Pathway Analysis (IPA)^32^. All pathways were illustrated through IPA^32^. Only SVs with at least one affected cancer cat were evaluated.

### Statistics and reproducibility

Assembly statistics for each reference genome were obtained from the NCBI website. BUSCO statistics for the fishing cat reference was determined using BUSCO (version 4.1.2_cv1)^22^. All SNP and Indel statistics from the variant calling pipeline were obtained using BCFTools stats (version 1.14)^55^. All sample requirements for DNA or RNA isolation were followed in accordance with the following protocols: 10 × Genomics Demonstrated Protocol: DNA Extraction from Single Insects (10 × Genomics), Qiagen RNeasy Plus Universal Mini Kit (Qiagen), and Qiagen DNeasy Blood and Tissue Kit (Qiagen).

### Ethics approval and consent to participate

This study was conducted in accordance with the Association of Zoos and Aquariums fishing cat Species Survival Plan coordinator Tyler Boyd. All samples collected were from AZA Accredited facilities and through the Feline Genetics and Comparative Medicine Laboratory at the University of Missouri.

### Supplementary Information

Supplementary Information 1.

Supplementary Information 2.

## Data Availability

All raw and processed data for this study are available at NCBI BioProject under accession PRJNA815338.

## Code availability

Scripts used for this study are available at the following GitHub repositories: https://github.com/esrice/hic-pipelinehttps://github.com/WarrenLab/purge-haplotigs-nfhttps://github.com/WarrenLab/agptools.
