# Supplementary material for: A chromosome-scale fishing cat reference genome for the evaluation of potential germline risk variants
Source: Sci Rep. 2024 Apr 5;14:8073. doi: 10.1038/s41598-024-56003-7 (PMC10997796; doi:10.1038/s41598-024-56003-7)
Supplement: Supplementary file 2 — Supplementary Information 2. [file 41598_2024_56003_MOESM2_ESM.pdf]

a b
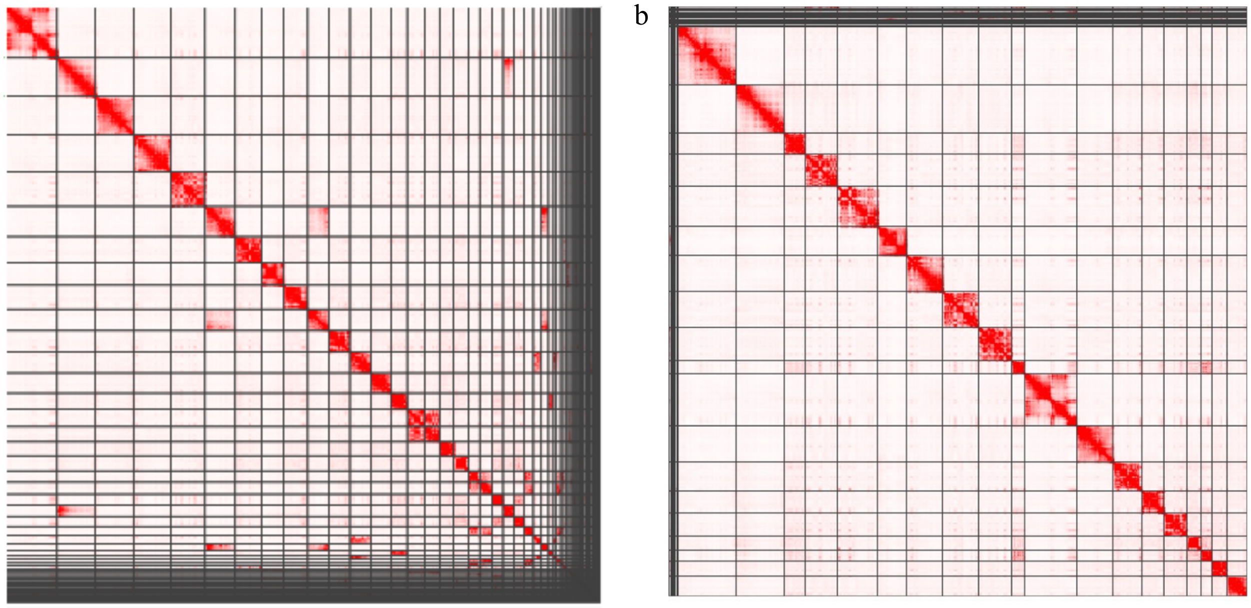

Supplementary Figure 1. Fishing cat Hi-C mapping outputs.
a) Depicted here is the Hi-C image of the pre-corrected fishing cat scaffold level assembly. **b)** The final Hi-C image of the 19 fishing cat chromosomes.

a b

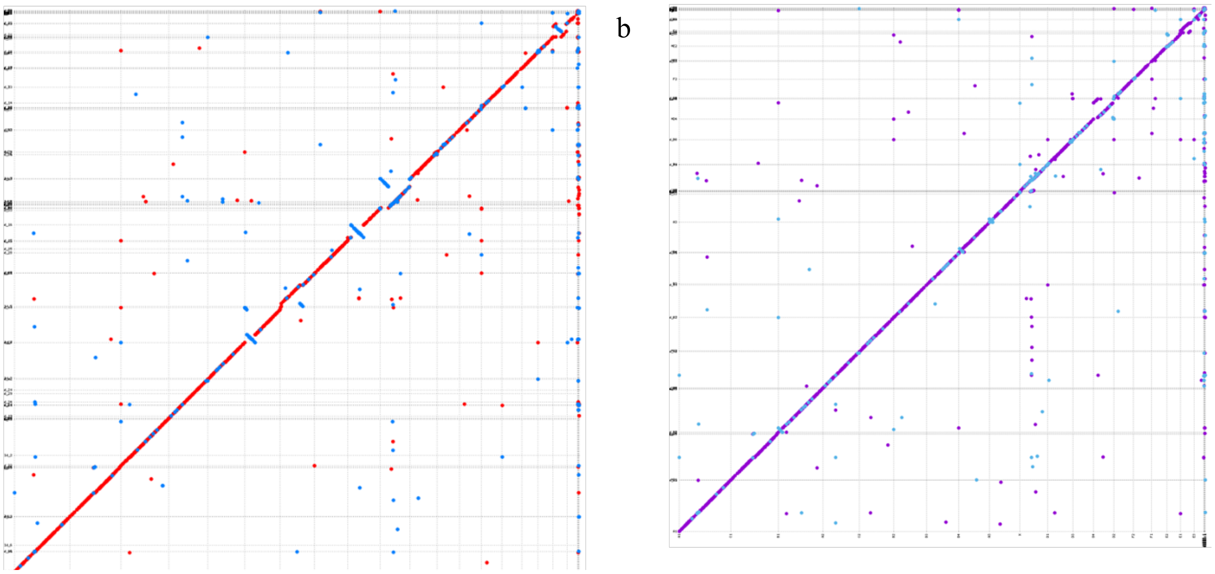

Supplementary Figure 2. Scaffold correction using alignments to a similar feline genome.
a) Pre-corrected MashMap dot plot comparing the fishing cat scaffold assembly (y) to the domestic cat assembly (x). Negative alignment correlations are blue and positive alignment correlations are red. **b)** Finalized MashMap output of the fishing cat assembly (y) compared to the domestic cat assembly (x). Negative correlation alignments are indicated in blue and positive alignment correlations are purple.

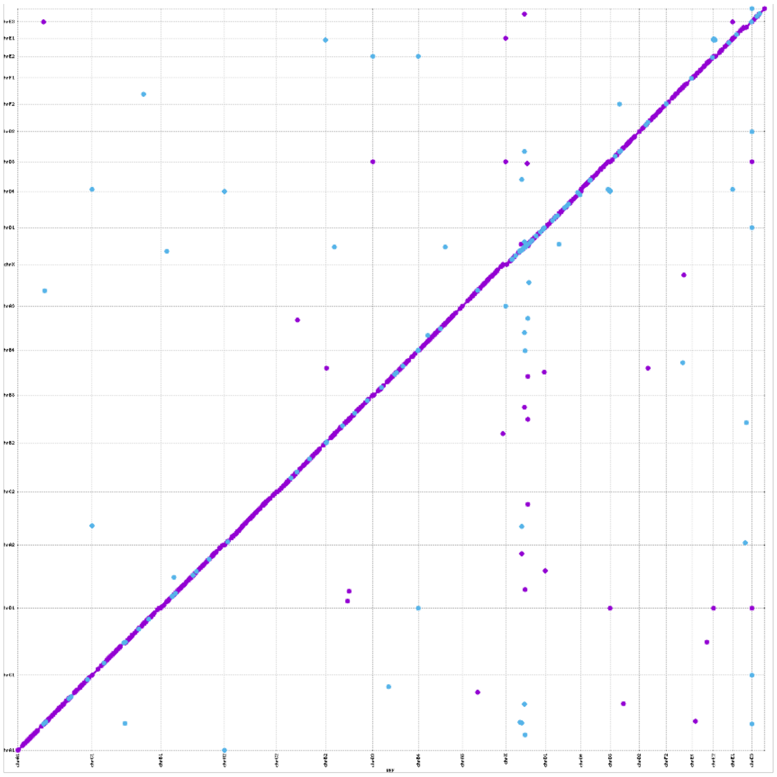

Supplementary Figure 3. Alignment relationship between the Asian leopard cat and fishing cat genome.
Above is the MashMap output comparing the fishing cat chromosome level assembly (y) to the Asian leopard cat assembly (x). Negative alignment correlations are indicated in blue and positive correlations are indicated by purple.

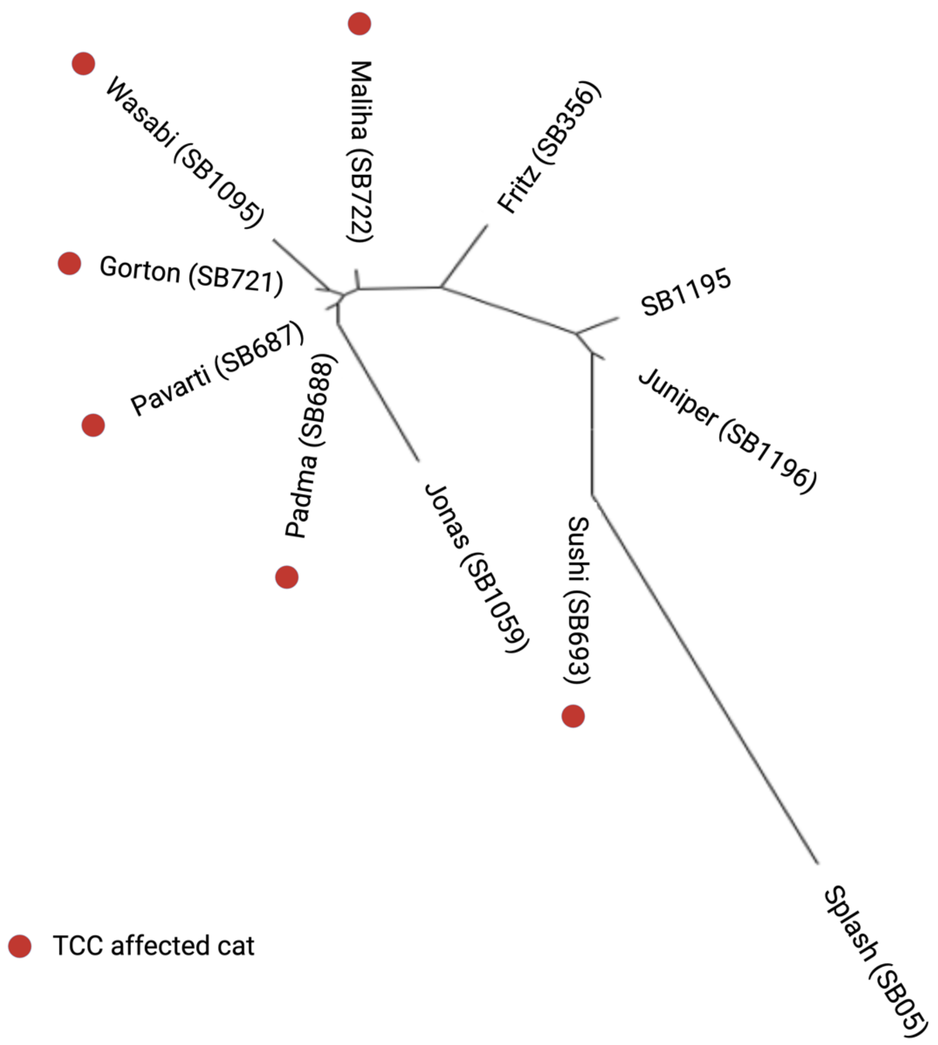

Supplementary Figure 4. Cohort neighbor joining tree.
Illustrated here is a phylogeny tree depicting the shared relationships among the fishing cat cohort. Name and studbook numbers are indicated. Affected cancer individuals are indicated by a red dot.

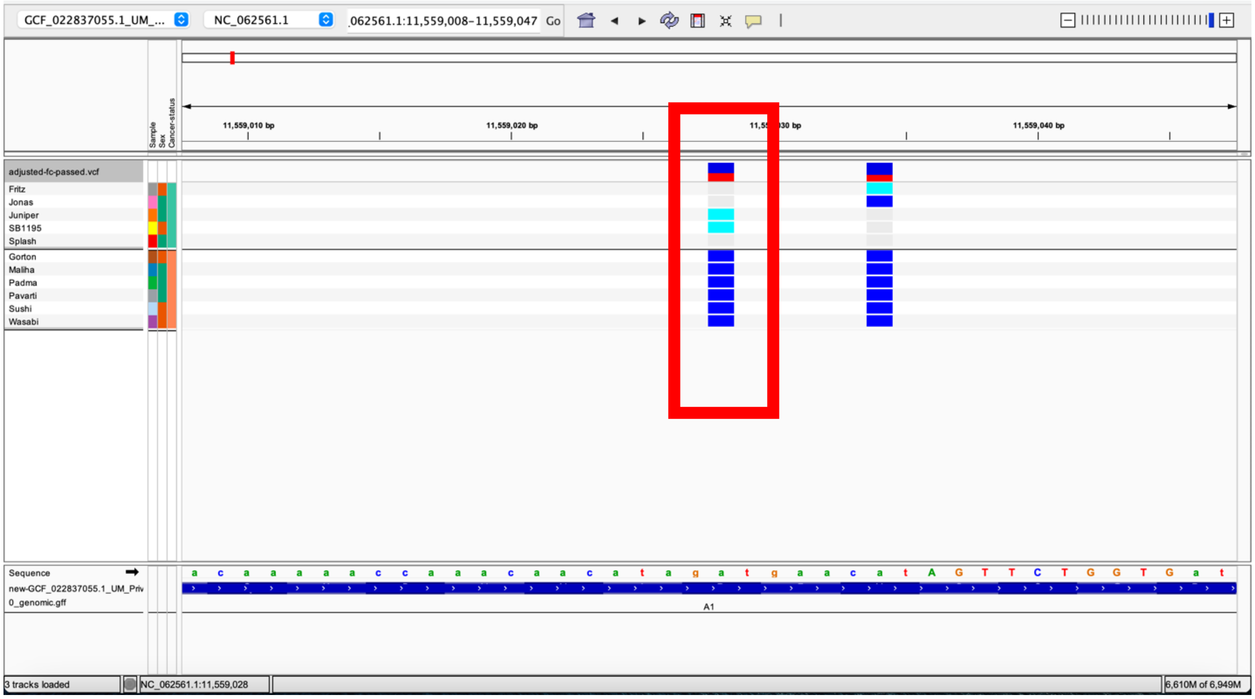

Supplementary Figure 5. Missense *BRCA2* position in cancer affected cats. The above IGV Frame illustrates the missense *BRCA2* position, indicated by the red box, with a heterozygous genotype that uniquely present only in all cancer cats.

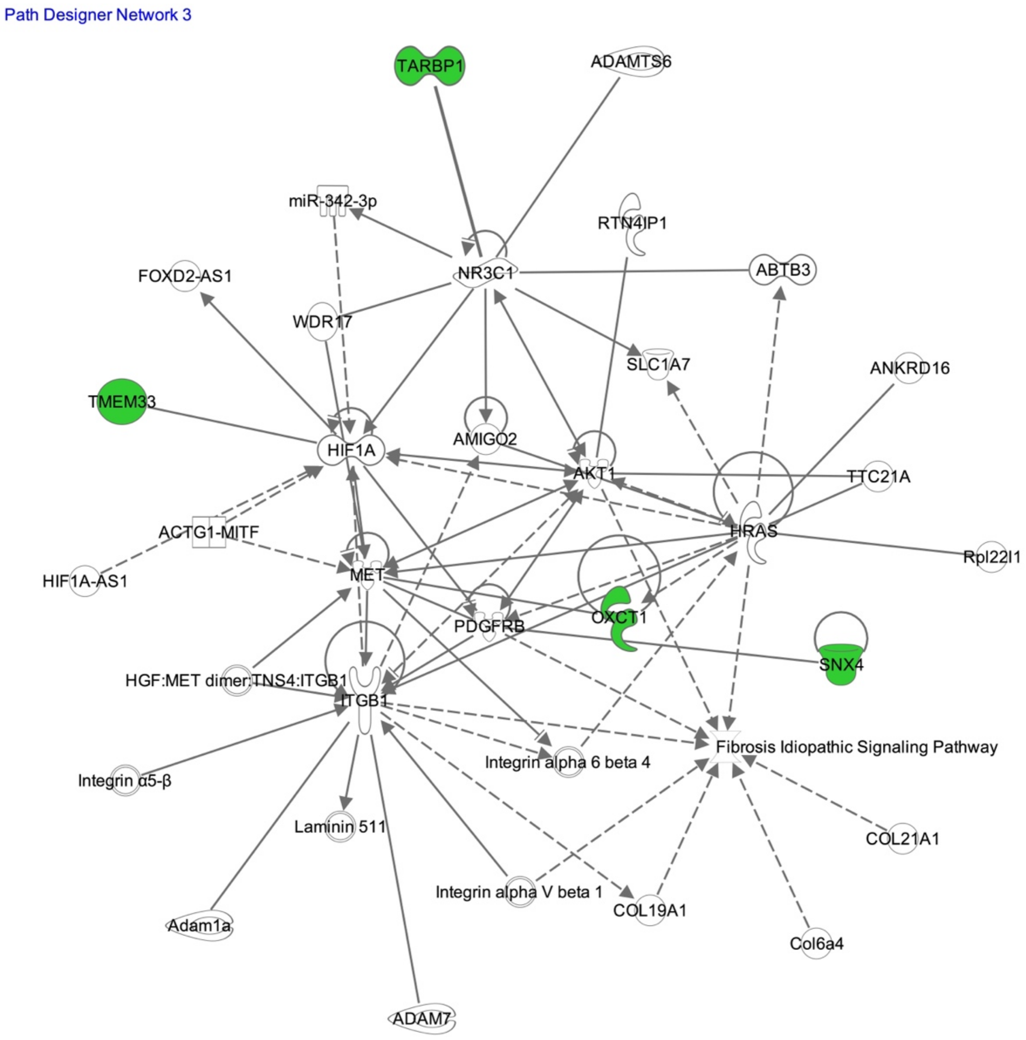

© 2000-2023 QIAGEN. All rights reserved.

Supplementary Figure 6. Tumorigenesis pathway of genes with SV affected coding regions. The above diagram illustrates a tumorigenesis pathway containing genes (highlighted in green) with coding regions affected by SVs in the fishing cat cohort. Only genes with at least one cancer cat containing the SV of interest are highlighted.
